# Chasing the Genetic Landscape Beyond the Pulmonary Veins

**DOI:** 10.1016/j.jacadv.2026.102940

**Published:** 2026-06-17

**Authors:** James P. Pirruccello, Shinwan Kany

**Affiliations:** a Division of Cardiology, University of California-San Francisco, San Francisco, California, USA; b Department of Cardiology, University Heart and Vascular Centre, Hamburg, Germany

**Keywords:** atrial fibrillation, genetics, genome-wide association study

Catheter ablation is firmly established as a frontline therapy for paroxysmal atrial fibrillation (AF).^2^^,^^3^ The standard procedural approach is conceptually straightforward: we electrically isolate the pulmonary veins as elegantly demonstrated by Haïssaguerre et al^4^ in 1997. However, clinical reality in AF care is rarely so simple. Recent trials evaluating pulsed field ablation for AF exhibit highly optimized technical success for pulmonary vein isolation, yet recurrence rates remain frustratingly high with 30% to 50% within 12 months.^5^, ^6^, ^7^

Despite these advances, clinical reality proves that postprocedural recurrences remain a persistent issue. Drivers of postablation recurrence are sometimes identified as nonpulmonary vein (non-PV) triggers which are ectopic foci arising from structures like the vein of Marshall, the superior vena cava, crista terminalis, coronary sinus, or the left atrial appendage among other locations. Although operators routinely chase these extrapulmonary foci during repeat procedures, predicting their presence before the index ablation remains a significant clinical challenge.^8^

Evaluating whether non-PV triggers cause AF recurrence after paroxysmal AF ablation requires untangling technical failure from true biological variation.

Currently, non-PV triggers are unmasked largely through aggressive pharmacological provocation during the electrophysiology study. If we could identify patients predisposed to non-PV triggers preprocedurally, we could tailor ablation strategies, perhaps deploying different mapping tools or adopting a more extensive ablation lesion set from the outset. Although specific patient factors such as female sex offer faint clues, robust predictive markers remain elusive.^9^

In this issue of *JACC: Advances*, Okamura et al^1^ offer a new biological perspective on this problem by approaching it through a genomic lens. They hypothesize that distinct genetic substrates drive the development of non-PV triggers. To test this, the authors conducted a genome-wide association study within a highly characterized cohort of 1,091 Japanese patients undergoing their first AF ablation. Rather than comparing AF cases to healthy controls, they used a case-only design to identify drivers of non-PV triggers. Patients with inducible non-PV triggers (12.5% of the discovery cohort) were compared directly against those with PV-only triggers.

The main discovery is that of a genome-wide significant single-nucleotide variant on chromosome 3 (rs117203318): patients harboring the minor allele of this variant had over 5-fold higher odds of being in the non-PV trigger group. Although there was no validation external to the single center where the study was conducted, the authors performed robust internal validation through a temporally separated cohort that used a different sequencing technology to confirm the association, an approach that eliminated several possible confounders. The polymorphism is nearly absent outside East Asian populations, which highlights the scientific value of sampling broadly from human genetic diversity, while also showcasing the challenges of post-genome-wide association study analyses: this noncoding polymorphism may well influence tissue expression of a nearby gene, but this cannot be detected in expression atlases derived from largely EUR-ancestry individuals who are monomorphic for this variant.

As the authors rightly note, a central uncertainty around the interpretation of this finding is whether rs117203318 influences non-PV triggered AF, or instead influences the electrophysiologic test that is used to categorize patients. The absence of the variant in a recent multiancestry meta-analysis of AF cases and controls—which incorporated a large number of EAS-ancestry participants—points away from an influence on AF biology.^10^ This interpretation has a material impact on the clinical consequences of these findings: if rs117203318 is a marker of AF biology, then the effector gene becomes a new therapeutic target for non-PV-triggered AF, with *SERP1* as a nearby and mechanistically plausible candidate. *SERP1* is involved in stabilizing proteins during endoplasmic reticulum stress, which has been linked to AF in cell models.^9^ But if the variant influences the test used to distinguish PV from non-PV triggers, then the clinical takeaway may instead come through an improved understanding of the genetic drivers of responsiveness to electrophysiologic testing. The collected data may offer the opportunity to quantitatively analyze the differences in electroanatomical mapping between carriers and noncarriers of the rs117203318 minor allele, which could be informative about the biological underpinnings of these findings. Future work is needed to link the variant to its effector gene and to understand the molecular biology driving the findings.

Perhaps these limitations also show the way forward in our efforts to understand the etiology of AF not mediated by PV triggers. Genetics may help us to find our way, but precise phenotyping using currently available tools in the electrophysiology lab might provide a map to identify patients benefiting from a different catheter ablation approach beyond pulmonary vein isolation.

Ultimately, we are not yet at the stage of genotyping patients in the office before catheter ablation. However, Okamura et al^1^ provide a valuable mechanistic puzzle piece to our understanding. By linking a specific genetic locus to an adverse electrophysiological phenotype, they remind us that AF is not a monolithic disease and the pulmonary veins may not be the end of the road. Recognizing the genetic heterogeneity of AF and electrophysiologic testing is an essential first step toward truly personalized arrhythmia management.

## Funding support and author disclosures

Dr Pirruccello is supported by the 10.13039/100000050NIH/NHLBI via K08HL159346 and R01HL178603. Dr Kany is supported by the Walter Benjamin Fellowship from the 10.13039/501100001659Deutsche Forschungsgemeinschaft (521832260).
